# Supplementary material for: MetaRibo-Seq measures translation in microbiomes
Source: Nat Commun. 2020 Jun 29;11:3268. doi: 10.1038/s41467-020-17081-z (PMC7324362; doi:10.1038/s41467-020-17081-z)
Supplement: Supplementary file 10 — Supplementary Data 7 [file 41467_2020_17081_MOESM10_ESM.zip › File2/Confidence_High_Taxonomy/425704_out.krona.html]

Javascript must be enabled to view this page.

members
magnitude
magnitudeUnassigned
count
unassigned
taxon
rank

425704\_out

23

23
2
superkingdom

phylum
976
23

23
200643
class

23
171549
order

23
815
family

816
genus
23

4
species
1262735

SRS015590\_contig\_number\_12690SRS015663\_contig\_number\_27344SRS105082\_contig\_number\_635SRS143876\_contig\_number\_35039

13

SRS014415\_contig\_number\_1753SRS015782\_contig\_number\_35452SRS016018\_contig\_number\_14491SRS019178\_contig\_number\_358SRS019285\_contig\_number\_156SRS023715\_contig\_number\_25339SRS051031\_contig\_number\_12094SRS077589\_contig\_number\_5816SRS1041091\_contig\_number\_contig-100\_1688.108459SRS1041138\_contig\_number\_4348SRS1041157\_contig\_number\_916SRS143181\_contig\_number\_contig-100\_142.142SRS893285\_contig\_number\_74
species
310297

1
species
47678

SRS043841\_contig\_number\_258

3

SRS011302\_contig\_number\_contig-100\_3173.150681SRS078242\_contig\_number\_contig-100\_2318.142970SRS147271\_contig\_number\_30997
species
1263052

species
817

SRS147653\_contig\_number\_171
1


SRS064232\_contig\_number\_3703
species
1263044
1
